# Structure of a *Thermobifida fusca* lytic polysaccharide monooxygenase and mutagenesis of key residues

**DOI:** 10.1186/s13068-017-0925-7

**Published:** 2017-11-30

**Authors:** Nathan Kruer-Zerhusen, Markus Alahuhta, Vladimir V. Lunin, Michael E. Himmel, Yannick J. Bomble, David B. Wilson

**Affiliations:** 1 Department of Molecular Biology and Genetics, Cornell University, Ithaca, NY USA; 2 National Renewable Energy Laboratory, Golden, CO USA

**Keywords:** *Thermobifida fusca*, Biofuels, Biomass degrading enzymes, LPMO, Cellulose, Oxidative chemistry

## Abstract

**Background:**

Auxiliary activity (AA) enzymes are produced by numerous bacterial and fungal species to assist in the degradation of biomass. These enzymes are abundant but have yet to be fully characterized. Here, we report the X-ray structure of* Thermobifida fusca* AA10A (TfAA10A), investigate mutational characterization of key surface residues near its active site, and explore the importance of the various domains of* Thermobifida fusca* AA10B (TfAA10B). The structure of TfAA10A is similar to other bacterial LPMOs (lytic polysaccharide monooxygenases), including signs of photo-reduction and a distorted active site, with mixed features showing both type I and II copper coordination. The point mutation experiments of TfAA10A show that Trp82 and Asn83 are needed for binding, but only Trp82 affects activity. The TfAA10B domain truncation mutants reveal that CBM2 is crucial for the binding of substrate, but that the X1 module does not affect binding or activity.

**Results:**

In TfAA10A, Trp82 and Asn83 are needed for binding, but only Trp82 affects activity. The TfAA10B domain truncation mutants reveal that CBM2 is crucial for substrate binding, but that the X1 module does not affect binding or activity. The structure of TfAA10A is similar to other bacterial lytic polysaccharide monooxygenases with mixed features showing both type I and II copper coordination.

**Conclusions:**

The role of LPMOs and the variability of abundance in genomes are not fully explored. LPMOs likely perform initial attacks into crystalline cellulose to allow larger processive cellulases to bind and attack, but the precise nature of their synergistic behavior remains to be definitively characterized.

**Electronic supplementary material:**

The online version of this article (doi:10.1186/s13068-017-0925-7) contains supplementary material, which is available to authorized users.

## Background

Cellulosic biomass is a promising source of carbon for renewable fuels and chemicals. Biomass feedstocks can undergo enzymatic deconstruction to their component sugars, which can be used for a variety of bioprocesses. The economic feasibility of cellulosic biofuels is limited by substrate recalcitrance, the native physical properties of plant tissue and cell walls that limit the efficiency of sugar release. Cellulolytic bacteria and fungi overcome biomass recalcitrance by secreting complex enzyme mixtures, which can be optimized for industrial application. Novel components that catalyze biomass saccharification are being investigated to optimize commercial enzyme preparations and, therefore, enable economic feasibility of second generation fuels and renewable chemicals.

Cellulose is a semicrystalline matrix of anhydro-β-d-glucose linked by β-1,4-glycosidic bonds forming polysaccharide chains [1]. Cellulose resists depolymerization by hydrolytic cellulases because of its insolubility in water, highly crystalline structure, and surface complexity. Glycoside hydrolases (comprising 135 CAZy families [2]) perform most of the saccharification of biomass in microbial secretomes and commercial preparations. Cellulose degrading enzymes are found primarily in GH families 1,3, 5, 6, 7, 8, 9, 12, 44, 45, 48, 51, 74, and 124. Lytic polysaccharide monooxygenases (LPMOs) are auxiliary activity enzymes that also attack cellulose, as well as other polysaccharides, using an oxidative mechanism [3]. LPMOs are synergistic with hydrolytic cellulases, and significantly improve digestion by industrial cellulase preparation [4]. LPMO genes are abundant in both fungal and bacterial genomes, and multiple genes are often present in fungal genomes. LPMOs are compact globular enzymes that lack a substrate binding cleft or tunnel, and instead have a copper atom containing active site located near a planar binding surface. In the presence of a reducing agent and oxygen, LPMOs attack the surface of crystalline cellulose [5]. In contrast to processive cellulases, such as those found in GH families 6, 7, and 48, this mechanism avoids the search for an exposed cellulose chain and the slow process of positioning a cellulose chain end into a tunnel active site.

Oxidative activity on crystalline cellulose by LPMOs was first demonstrated for the LPMOs of *Serratia marcescens* [6]. The LPMO oxidative cleavage mechanism involves the creation of an oxygen radical from molecular oxygen, likely a Cu(II)-oxyl species, which abstracts hydrogen and hydroxylates the substrate [7]. Cellulose and chitin active LPMOs can target either side of the β-glycosidic bond. LPMOs that create oxidized products at glucose C1 are classified as type I; those that attack the C4 position are classified as type II, and those that have both activities are classified as type III [8–10]. *Thermobifida fusca*, a model cellulolytic bacterium, secretes two LPMOs, the type I (*Tf*AA10B) and type III (*Tf*AA10A) [11–13]. The C1 oxidized products of type I and type III LPMOs are *δ*-1,5 lactone sugars of varying lengths (that form aldonic acids when hydrated), and the C4 oxidized products of type II and type III LPMOs are 4-ketoaldoses (that are hydrated to form gemdiols) [14]. Oxidative cleavage results in an altered cellulose substrate that is more easily degraded by hydrolases. For example, *Tf*AA10A stimulates the activity of the processive exocellulase, TfCel48A [15].

The structures of several bacterial AA10 LPMOs have been solved, revealing common structural attributes that affect activity [16]. The first LPMO structure to be solved was the chitinolytic LPMO SmCBP21 and more recently several bacterial LPMOs acting on cellulose have been investigated [8, 17, 18]. These structures share a common immunoglobulin-like β-sandwich core fold, a flat binding surface, and a conserved *N*-terminal histidine. These conserved structural features are similar for LPMOs active on a range of insoluble substrates including chitin, indicating a common strategy for binding and active site positioning. The planar binding surface contains conserved polar residues, which function in binding to planar carbohydrate substrates. The structurally conserved copper coordination site positions the copper atom in proximity to the scissile carbohydrate bond and preserves the correct copper redox state [13]. The domain architecture of LPMOs is also shared across species, often with the LPMO catalytic domain (always at the *N* terminus due to the absolutely conserved *N*-terminal histidine involved in copper chelation) alone or attached to a binding domain.

In this work, we report an additional bacterial AA10 LPMO structure, AA10A from *Thermobifida fusca*, obtained using X-ray diffraction. The activity of *Tf*AA10A (formerly E7) has received considerable attention recently [12, 13]. To explore the mechanism used by LPMOs to bind and perform oxidative cleavage of crystalline substrates, we characterized *Tf*AA10A surface residue mutants and *Tf*AA10B domain truncations. The results obtained indicate that both polar and aromatic residues on the surface play critical roles for binding and activity; and that the CBM2 domain contributes significantly to *Tf*AA10B binding and activity.

## Methods

### Mutagenesis

*Tf*AA10A and *Tf*AA10B (formerly E7 and E8) were cloned in pET26b+ to replace the endogenous signal peptide with the PelB leader sequence. Mutants of *Tf*AA10A and *Tf*AA10B were created using the QuickChange II XL Site-Directed Mutagenesis Kit (Agilent), following established protocols. The *Tf*AA10B domain deletion construct was made by introducing a HindIII cut site to replace the X1 domain with a two residue (LE) linker sequence. All construct sequences were validated, expressed, and purified using established protocols [11]. Concentration of purified proteins was determined at OD280 using a calculated extinction coefficients [AA10A: 3.2461 (mg/mL)-1 cm-1; AA10B: 2.2488 (mg/mL)-1 cm-1] and samples were stored at − 80 °C.

### Crystallization

*Tf*AA10A crystals were initially obtained using sitting drop vapor diffusion and a 96-well plate with Crystal Screen HT from Hampton Research (Aliso Viejo, CA). Reservoirs contained 50 µL of well solution and drops had 0.2 µL of well solution and 0.2 µL of protein solution. A Phoenix crystallization robot (Art Robbins Instruments, Sunnyvale, CA) was used for setting up the screens. The best crystals were grown at 20 °C with 0.1 M Sodium acetate trihydrate pH 4.6, 20% v/v 2-Propanol and 0.2 M Calcium chloride dihydrate as the well solution. The protein solution that was used for crystallization contained 8.5 mg/mL of protein in 20 mM HEPES pH 7.5, 100 mM NaCl, 5% glycerol and 5% ethylene glycol.

### Data collection and processing

Both the native and potassium iodide (KI) soaked *Tf*AA10A crystals were flash frozen in a nitrogen gas stream at 100 K before data collection. Crystallization solution with 12.5% (v/v) ethylene glycol and glycerol each was used for freezing the crystal. Potassium iodide was introduced to the crystal by adding 0.5 M KI into the well solution and soaking the crystal in a 5 µL drop for 5 s before flash freezing. Data collection was performed using an in-house Bruker X8 MicroStar X-ray generator with Helios mirrors and a Bruker Platinum 135 CCD detector. Data were indexed and processed with the Bruker Suite of programs version 2011.2-0 (Bruker AXS, Madison, WI).

### Structure solution and refinement

The CCP4 package of programs [19], specifically SCALEPACK2MTZ, ctruncate, MTZDUMP, Unique, CAD, FREERFLAG and MTZUTILS, were used to convert intensities into structure factors and 5% of the reflections was flagged for Rfree calculations. The structure of *Tf*AA10A was solved using SIRAS with Crank2 [20, 21]. Buccaneer [22] was used to auto build the resulting partial model. Refinement and manual correction were performed using REFMAC5 [23] version 5.7.0029 and Coot [24] version 0.6.2. Phenix.refine version 1.10-2155 [25] was used for occupancy refinement followed by REFMAC5. The MOLPROBITY method [26] was used to analyze the Ramachandran plot and root mean square deviations (rmsd) of bond lengths and angles were calculated from ideal values of Engh and Huber stereochemical parameters [27]. Wilson B-factor was calculated using CTRUNCATE version 1.17.7. The data collection and refinement statistics are shown in Table 1.

**Table 1:** X-ray data collection and refinement statistics. Statistics for the highest resolution bin are in parenthesis

Data collection	
Space group	P 32 2 1
Unit cell, Å, °	*a* = 77.23, *b* = 77.23, *c* = 66.753 *β* = 122.35
Wavelength, Å	1.54178
Temperature (K)	100
Resolution, Å	25.0–1.99 (2.09–1.99)
Unique reflections	28,968 (3560)
*R*_int_^a^	0.155 (0.622)
Average redundancy	11.9 (3.4)
<I >/<σ(I)>	10.6 (1.4)
Completeness, %	97.9 (90.1)
Refinement	
Resolution, Å	25–2.0 (2.05–2.0)
*R*/*R*_free_	0.164 (0.302)/0.229 (0.306)
Protein atoms	3126
Water molecules	367
Other atoms	42
RMSD from ideal bond length, Å^b^	0.016
RMSD from ideal bond angles, °^b^	1.648
Wilson *B*-factor	4.8
Average *B*-factor for protein atoms, Å^2^	21.8
Average *B*-factor for water molecules, Å^2^	29.2
Ramachandran plot statistics, %^c^	
Allowed	100%
Favored	97.1%
Outliers	0

^a^Rint = ∑| I – < I>|/∑|I| where I is the intensity of an individual reflection and < I > is the mean intensity of a group of equivalents and the sums are calculated over all reflections with more than one equivalent measured

^b^[27]

^c^[26]

### Structure analysis

Programs Coot and PyMOL (http://www.pymol.org) were used for comparing and analyzing structures. This structure has been deposited to the protein data bank (PDB; www.rcsb.org) with entry code 5UIZ.

### Substrates and reducing agent

Bacterial cellulose (BC), a gift from Monsanto, was washed and prepared as described previously [28]. The concentration was determined by dry weight using a vacuum oven, and it was stored at 4 °C in MilliQ water with 0.02% sodium azide to prevent microbial contamination. All activity assays contained reduced glutathione (Sigma) as a reducing agent to enable LPMO activity. Glutathione was stored dry at − 20 °C away from light, and a concentrated stock was prepared fresh for each assay by adjusting to pH 6.0 with sodium acetate.

### Binding assays

Binding affinity assays combined 1 μM of each LPMO variant with 2.5 mg/mL bacterial cellulose and 10 mM sodium acetate (pH 6.0) to a total volume of 160 μL. All samples were assembled in triplicate with representative buffer, substrate, and enzyme negative controls. Samples were sealed in 96-well Protein LoBind microplates (Eppendorf), and incubated at 50 °C with continuous horizontal shaking (160 RPM) for 16 h to ensure a binding equilibrium was reached. After incubation, samples were immediately centrifuged at 4000 RPM (3313xRCF) for 5 min at room temperature to precipitate the substrate. A 40 μL fraction of the supernatant was carefully removed and combined with 160 μL Quick Start Bradford reagent (Bio-Rad) in a 96-well microplate (Costar). Samples and standards were measured using a Synergy 4 plate reader (Biotek Instruments), and collected as the *A*_595_/*A*_450_ ratio to extend the sensitivity of the Bradford assay [29]. Unbound protein was quantified using a BSA standard curve, and extent of binding determined by comparison with enzyme only negative controls.

### Assay method

Cellulose digestion assays were assembled using 0.5 μM of WT or mutant LPMO, 5.0 mg/mL bacterial cellulose, 1 μM CuSO_4_, and 2 mM reduced glutathione to facilitate activity. To limit free radical reactions, each reaction contained 10 μg/ml catalase from *Aspergillus niger* (70 U, Sigma) that had been buffer exchanged thoroughly. Samples contained 50 mM sodium acetate (pH 6.0) in a final volume of 160 μL. All assays were run in triplicate, with representative controls and standards adjacent to sample wells. Plates containing kinetic time course samples were automatically removed at the designated intervals, transferred to a preheated PCR machine (MJResearch Inc.), heated to 100 °C for 5 min, and stored at room temperature until sample processing. Assay supernatant was separated from the insoluble substrate via centrifugation at 4000 RPM (3313xRCF) for 5 min before careful removal of supernatant for secondary hydrolysis. Secondary hydrolysis was performed to simplify quantification using Cel5A catalytic domain (50 μM) and Novo188 β-d-glucosidase (0.02 CBU, Novozymes) that had been buffer exchanged to remove most background signal.

### HPLC quantification

Samples from secondary hydrolysis were filtered through a 96 well 0.45 μm Supor^®^ filter (Pall) and were quantified using a Shimadzu Ultramate HPLC equipped with RID and UV detectors. An Aminex HPX-87H column (Bio-Rad) with a standard guard column was operated with isocratic flow at 0.6 mL/min with 0.005 M H_2_SO_4_ as eluent. 50 μL of each sample for HPLC detection was injected using a refrigerated autoinjector. Both neutral and oxidized sugars were detected with the RI detector, while oxidized sugars were detected at 200 nm in the UV channel [30]. Monosaccharide standards, glucose and gluconolactone (Sigma), were quantified during each run.

### Data processing

OriginPro 2016 (OriginLab Corp.) was used to process the raw data. A Gaussian fit was applied to chromatogram peaks after removal of buffer background to yield values of area under the curve. These values were compared to a linear standard curve to determine concentration. The fraction of oxidized product was determined by a standard curve of varying ratios, yielding the concentration of each product. The extent of digestion was determined by comparison of the sum of monosaccharides released as initial anhydrous G1 based on dry weight determination. The kinetic time course data were plotted as % digestion vs time, and nonlinear kinetic parameters were determined based on the two-parameter model using existing protocols [15].

## Results and discussion

### The crystal structure of *T. fusca* AA10A

**Fig. 1:**
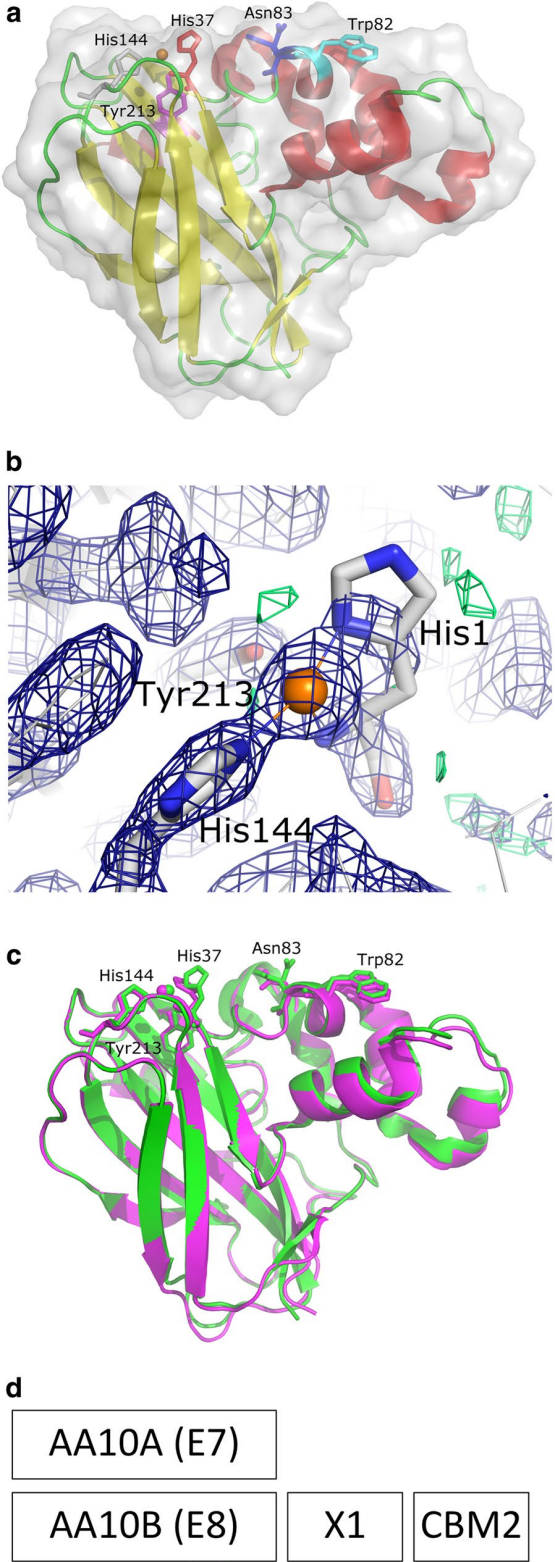
Structure of *T. fusca* AA10A and the domain arrangement of *T. fusca* LPMOs. **a** Overall structure of *Tf*AA10A shown as a ribbon with red α-helices, yellow β-sheets and green loops with a surface representation positioned to emphasize the planar binding surface. Position of surface mutant residues is shown as sticks in red (His37), magenta (Tyr213), blue (Asn83, both conformations), and cyan Trp82). An orange ball depicts the copper atom and to complete the active site His144 is shown as gray sticks. **b** Electron density figure showing the active site of *T. fusca* AA10A chain A. Residues are shown as sticks with gray carbons, blue nitrogens and red oxygens. The copper atom is shown as an orange ball. The 2Fo-Fc map is shown as a dark blue mesh and has been calculated at 1.2 sigma 7.5 Å around the copper atom. The Fo-Fc map is green at 3.2 sigma and has been rendered 7.5 Å from the copper atom. Both maps were calculated using REFMAC5 and MAPMASK with the CCP4 interface [19, 23]. **c** *Tf*AA10A and *Streptomyces coelicolor* lytic polysaccharide monooxygenase superimposed. *Tf*AA10A is shown as a green ribbon with the discussed residues labeled and shown as green sticks. *Streptomyces coelicolor* lytic polysaccharide monooxygenase is shown in magenta. **d** Domain arrangement of *T. fusca* LPMOs showing *Tf*AA10A and the multiple *Tf*AA10B domains

The structure of *Tf*AA10A was refined to a resolution of 2.0 Å with *R* and Rfree of 0.167 and 0.233, respectively. There are two molecules in the asymmetric unit with two copper atoms and multiple iodides (Fig. 1a). It has an Ig-like β-sandwich fold with a copper ion bound on the active site. In both molecules, the copper ion is only partially present with incomplete coordination likely due to low occupancy partial conformations at the active site that cannot be modeled properly. This is common with AA10 LPMOs [16]. The His37 forming the histidine brace and residues discussed in this manuscript, His144, Tyr213, Trp82 and Asn83, can be seen in electron density (Fig. 1b). The His37 residues of both chain *A* and *B* have somewhat lowered occupancy and the electron density for the His37 in chain *A* is weaker for part of the imidazole ring indicating conformational variability. The Asn83 residue adjacent to the copper active site is highly conserved, with two alternating conformations in chain *A* and atomic thermal displacement parameters indicating high mobility of the side chains. The adjacent aromatic residue present on the *Tf*AA10A binding surface, Trp82, does not have high conservation. It has been suggested that this aromatic residue performs a substrate targeting role rather than increasing total binding affinity, based on models of the *Phanerochaete chrysosporium* GH61D AA9 LPMO [31]. The structure of *Tf*AA10A has been deposited to the protein data bank (PDB; www.rcsb.org) with entry code 5UIZ.

### Structural similarity of *T. fusca* AA10A

The structure of *Tf*AA10A shares many features with other recently crystallized AA10 LPMOs [16]. Eight similar clusters with 40% sequence identity clustering were obtained from the protein data bank (PDB; www.rcsb.org) using the jFATCAT-rigid algorithm [32, 33]. From these proteins, the most similar was clearly the *Streptomyces coelicolor* lytic polysaccharide monooxygenase (PDB code 4OY6) [13], which has a sequence identity of 70% and C*α* root mean square deviations of 0.66 Å, showing that the overall backbone is the same. The other seven structures had sequence identities below 30%. Closer comparison between the *Streptomyces coelicolor* lytic polysaccharide monooxygenase and *Tf*AA10A reveals almost identical backbone and His144, Tyr213, Trp82 and Asn83 at the same locations and conformations (Fig. 1c).

The sequence of *Tf*AA10A is similar to the catalytic domain of *Tf*AA10B, sharing 33% residue identity, with the exception of an additional stretch of seven amino acids present in *Tf*AA10B. *Tf*AA10A does not have any auxiliary domains unlike *Tf*AA10B which also includes a CBM2 and an X1 (Fn3-like) domain (Fig. 1d). An interesting feature of *Tf*AA10A is the axial position of the copper coordination sphere, which is tyrosine. In most AA10 LPMOs and in *Tf*AA10B, a phenylalanine occupies this position.

### Copper coordination

**Fig. 2:**
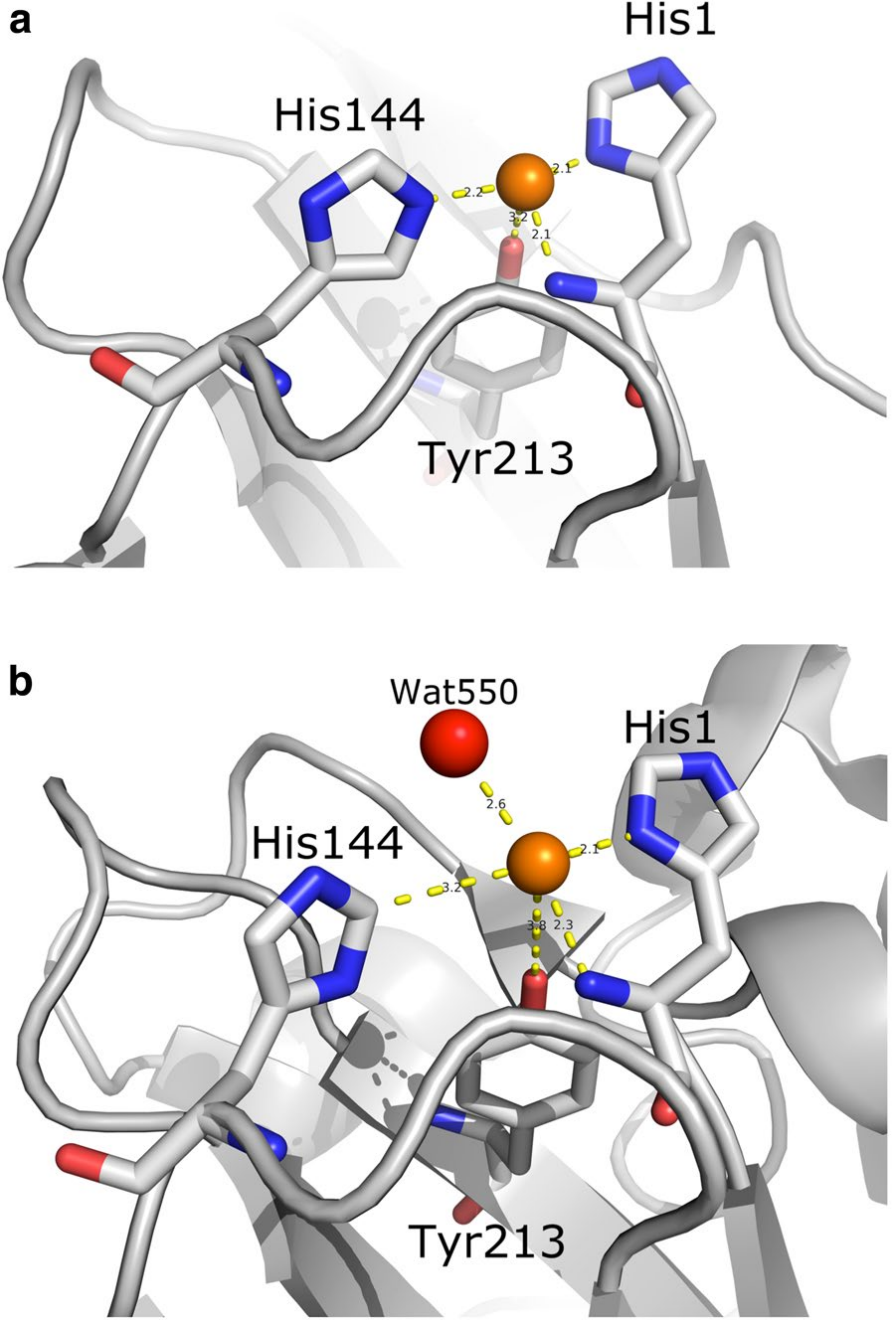
The active site of *T. fusca* AA10A with coordination distances. **a** Chain A. **b** Chain B. Residues are shown as sticks with gray carbons, blue nitrogens and red oxygens. The copper atom is shown as an orange ball

The presence of copper at the *Tf*AA10A active site is in agreement with similar structures and EPR results supporting copper as the essential metal [13]. The coordination of the *Tf*AA10A copper is similar to other structures, supporting a common mechanism of copper coordination (Fig. 2 a, b). A histidine brace, a conserved feature of LPMOs coordination with copper [8, 9, 34–36], facilitated by the *N*-terminal histidine 37 can be clearly observed in *Tf*AA10A. The two copper atoms in the asymmetric unit are both partially occupied after occupancy refinement. The copper atom at the active site of chain A is 50% occupied and the one with chain B has occupancy of 31%. The active site of chain A has type I copper coordination in agreement with previous studies [16] while the more distorted active site of chain B includes some features similar to type 2 with an equatorial water (wat550) in contact with the copper (Fig. 2a, b). Clearly, both active sites have been photo-reduced by X-ray radiation during data collection supporting the assumption that copper(II) is the catalytically competent state. However, the active site of chain B cannot be used for more detailed analysis of the active site coordination due to distortion. Specifically, the occupancy of the copper atom at this site is low (31%) after occupancy refinement, the distance to His144 is too long and in its main conformation His144 is hydrogen bonded to water 409 instead of coordinating with the copper atom (Fig. 2b).

### On measuring LPMO kinetics: reducing agents

LPMO reaction kinetics includes all the complexity of cellulase kinetics acting on changing recalcitrant substrates, but with additional challenges for assay design and product detection. Multiple small molecule reducing agents have been found to enable LPMO activity [37–40]. Ascorbate has several disadvantages that complicate activity quantification. Its major oxidized form, dehydroascorbate, degrades into a complex mixture of products when boiled, which co-elute with oxidized monosacharides. Ascorbate is also spontaneously oxidized by copper, making it a less suitable reducing agent [38]. As a source of reducing power, glutathione (GSH) is capable of single and double electron transfer [39]. GSH appears to have the same protective role in kinetics assays, as it does in cells, helping to maintain enzyme activity in the presence of oxygen radicals. The majority of glutathione forms a stable structure (GSSG) after oxidation that does not produce degradation products after boiling.

### On measuring LPMO kinetics: unreliable kinetics

The wide product distribution created by pseudo-random cleavage makes the kinetics of LPMOs difficult to measure accurately [9]. To alleviate this, an excess of β-d-glucosidase (0.02 CBU) was used in conjunction with the catalytic domain of TfCel5A (50 nM), an endocellulase capable of degrading longer oligosaccharides. Secondary hydrolysis was complete after overnight incubation based on the absence of neutral or oxidized oligosaccharide products. The β-d-glucosidase used had no effect on glucose, as all standards were incubated under similar conditions of secondary hydrolysis to confirm the absence of any background lactone signal. Lactonase, which catalyzes the hydrolysis of gluconolactone to gluconic acid, is present in Novozymes 188 [41]. With the presence of lactonase, it was assumed that all soluble lactones were hydrolyzed despite the slower rate of spontaneous lactone hydrolysis at lower pH. However, if C1 oxidation products remained unhydrolyzed, quantification would remain unchanged, as gluconolactone and gluconic acid produced identical standard curves (data not shown).

Catalase was included in LPMO reactions to prevent damaging free radical reactions and preserve activity [42]. The creation of peroxide side products in solution is a predicted mechanism through which LPMOs are inactivated over time [35]. Peroxides may reduce measured kinetic activity by destroying enzyme structure directly or through consuming the soluble reducing agent [42]. The inhibition of LPMO activity by catalase observed by others [43] was not observed, possibly due to differences between bovine and fungal catalase. Furthermore, in our hands catalase does not stimulate activity of LPMOs or hydrolytic cellulases directly. In the kinetic time course reactions presented here, both molecular oxygen and reducing agents are present in excess, necessitating the addition of catalase to protect LPMO activity.

### On measuring LPMO kinetics: product determination

HPLC determination of oxidized sugars using the Aminex HPX-87H was able to produce the ratio of C1 oxidized aldonic acids in samples (Additional file 1: Figure S1) based on previous work using commercial mixtures [30]. This detection approach enables quantification using standard saccharide HPLC equipment, but suffers from lower resolution and product detection limits compared to other methods. The gluconolactone standard curve is very linear (*R* ^2^ = 0.99) and serves as an effective standard for quantification of oxidized glucose products. This detection approach is only useful in cases where obtaining the product distribution is not necessary, as it requires complete secondary hydrolysis.

This determination of oxidized products from secondary hydrolysis of reactions containing LPMOs relies on detection of the carbonyl group. UV detection cannot directly measure the conversion to gemdiol forms, as alcohol groups do not absorb at 200 nm. A different approach is required to accurately quantify the net oxidized products of type III LPMOs like AA10A that produces both gluconic acids (type I) and 4-ketoaldoses (type II). Some 4-ketoaldose products become hydrolyzed to the gemdiol form and cannot be quantified using this method. The proportion of 4-keto AA10A products compared to gluconic acids is not known. Due to this characteristic, the number of C4 oxidative cleavage events may be underestimated for *Tf*AA10A and its mutants.

### *Tf*AA10A surface mutants: binding and activity on crystalline cellulose

Binding is essential for LPMO activity and thus to understand the mechanistic basis of LPMO activity, we must compare mutants with altered binding properties. Mutants of LPMO surface residues and domain architecture constructs help to reveal the mechanism of binding. Multiple residues on the *Tf*AA10A surface were mutated (H37A, W82A, N83A and Y213F; Fig. 1a), and constructs removing domains from *Tf*AA10B were compared in binding to crystalline cellulose (BC).

**Fig. 3:**
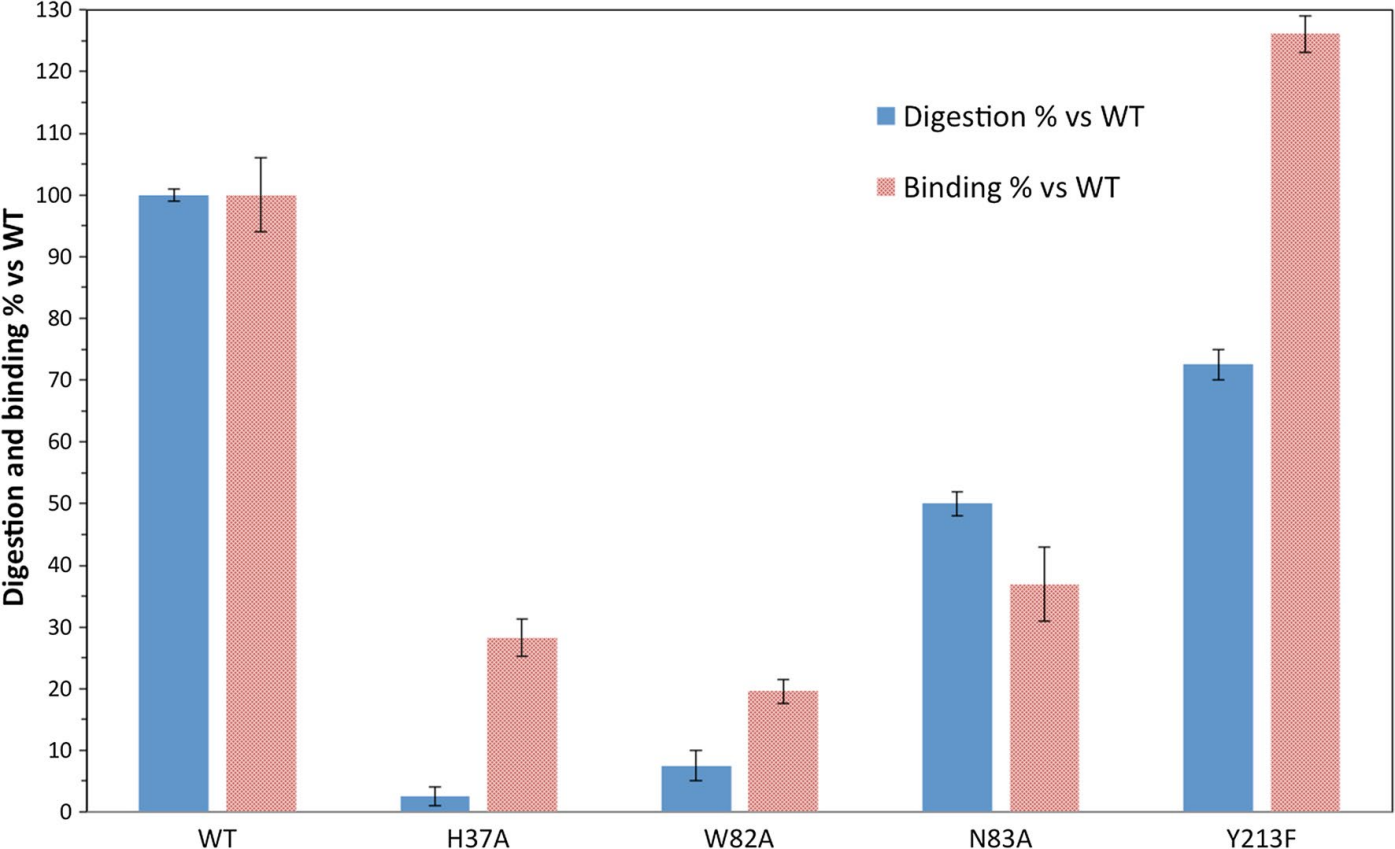
Binding and activity of *T. fusca* AA10A binding surface mutants. Digestion of 0.5 μM *Tf*AA10A incubated 2 h on 5.0 mg/mL BC, with total monosaccharide release compared with WT value. Extent of binding compared to WT as fraction of 1.0 μM enzyme lost from solution after binding equilibrium established after 16 h. Binding was measured in the absence of reducing agent. Samples were measured in triplicate, with error bars representing the replicate standard deviation

Mutation of conserved residues on the substrate binding surface had a significant effect on binding (Fig. 3). Compared to WT *Tf*AA10A, binding surface mutants showed decreased binding affinity when measured at 16 h after equilibrium had been established. The significant decrease in binding due to removal of the binding surface tryptophan, Trp82, and asparagine, Asn83, indicates that both residues play a critical role in binding to crystalline cellulose. This observation is in agreement with previous results that showed that mutating the aromatic tyrosine of SmCBP21 (in the same position as *Tf*AA10A Trp82) significantly decreases binding to chitin [17]. Removal of the *N*-terminal histidine residue (His37) eliminates the copper-histidine brace and consequently the binding of copper to the LPMO active site. This change greatly diminishes binding, supporting the significant role of the copper-histidine brace in mediating LPMO–substrate interaction. The change of *Tf*AA10A Tyr213 to phenylalanine increased binding and probably changes the coordination sphere of the copper to resemble *Tf*AA10B and other AA10 LPMOs [13, 44].

Furthermore, *Tf*AA10A surface mutants have significantly altered activity compared to WT *Tf*AA10A (Fig. 3). The H37A mutation eliminates essentially all oxidative activity relative to WT. This result is expected based on the important role of the His37 residue in providing the correct coordinating shell for the Cu atom within the active site. Also, the activity of W82A on PASC was significantly decreased relative to WT, to a similar degree to the loss of binding. The activity of the N83A mutant on PASC was much decreased relative to WT *Tf*AA10A, but significantly less than the W82 mutant. This result indicates the importance of this conserved polar residue for binding, but once bound, the substrate may need to be positioned correctly by W82 to enable activity. The Y213F mutation to match the residue in the axial position of the copper coordination sphere in *Tf*AA10B showed less change relative to WT *Tf*AA10A. The mutation to Y213F had 28% less activity compared to WT and 26% more binding. Lower but not eliminated activity from the Y213F mutation is in line with previous studies where this tyrosine was mutated to alanine [45]. The presence of an additional hydroxyl group from tyrosine affects the hydrogen bonding network and consequently positioning of the copper in *Tf*AA10A, but does not completely inhibit the activation of the copper to generate super-oxo species for catalytic attack. This observation is in agreement with previous electron spin resonance results exploring LPMO axial position occupancy [13].

The trend of *Tf*AA10A surface mutant activity largely matches the trend of substrate binding, with the exception of the N83A mutant where digestion is less affected compared to binding. Moreover, the differences between binding and activity may be relevant to understanding the structure–function relationship for this LPMO AA family. Binding and activity are not always directly coupled, based on results of the N83A mutant, where binding was greatly weakened but significant activity remained. The weak binding at equilibrium indicates that while the binding was not as stable, it existed long enough to position the LPMO active site over the substrate bond. The W82A mutant had a larger effect on activity than the N83A mutant, which supports a role for LPMO surface aromatics performing a critical role in substrate positioning; as well as lowering the binding energy [31].

### *Tf*AA10B domain mutants: binding and activity on crystalline cellulose

**Fig. 4:**
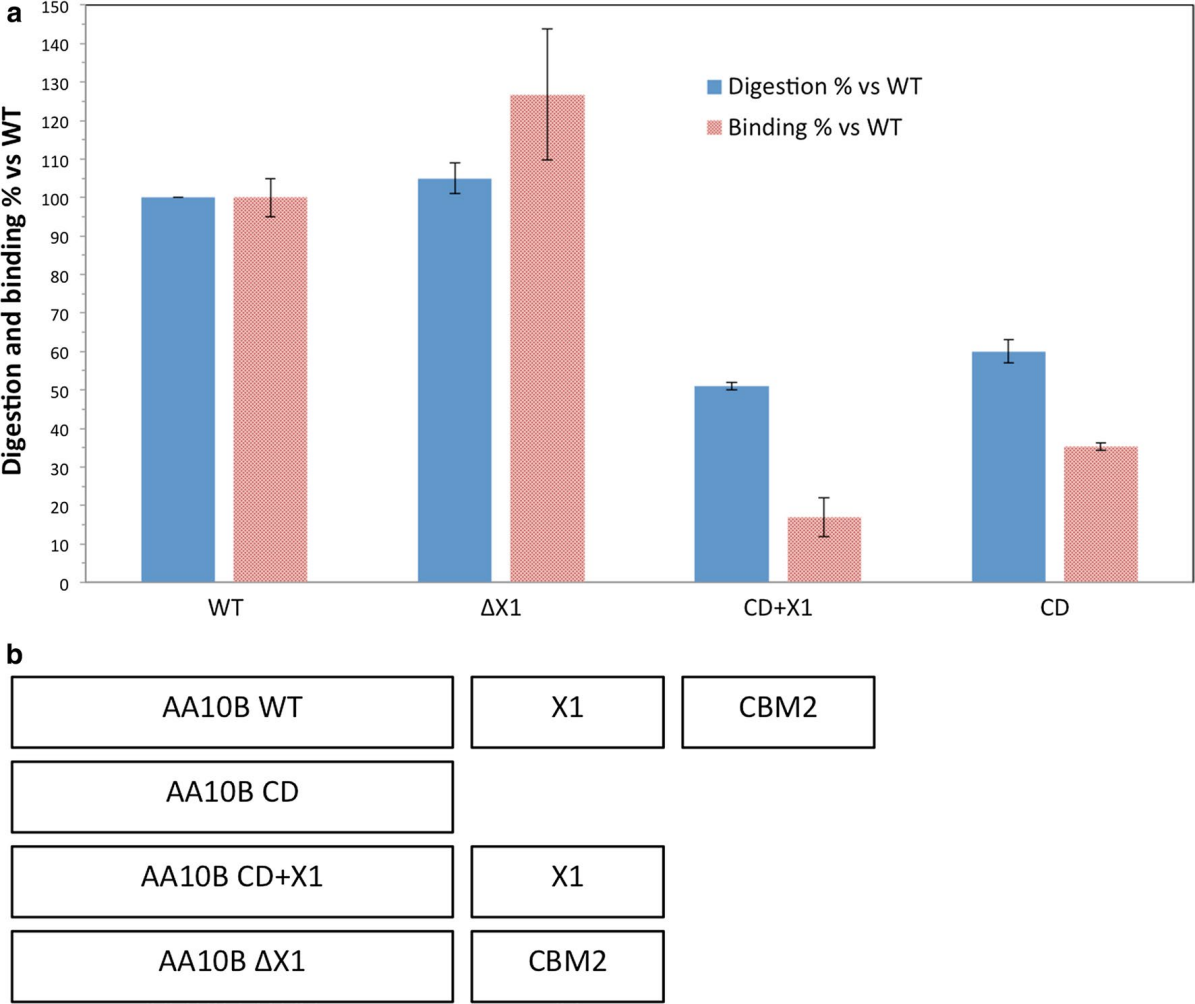
Binding and activity of *Tf*AA10B domain truncation mutants. **a** Digestion relative to WT measured as 0.5 μM LPMO on 5.0 mg/mL BC with 2 mM reduced glutathione after 2 h. As above, binding measured as 1.0 μM LPMO after 16 h incubation in the absence of reducing agent. Samples measured in triplicate, with error bars representing one standard deviation. **b** Domain architecture of constructs

**Fig. 5:**
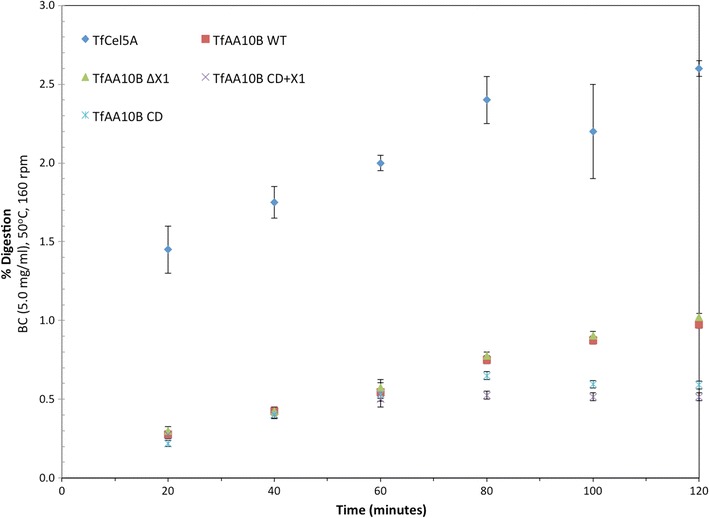
Activity of *Tf*AA10B truncation mutants compared with endocellulase *Tf*Cel5A. Time course reactions measured as 0.5 μM LPMO on 5.0 mg/mL BC with 2 mM reduced glutathione [15]

The most significant difference appears to be the removal of the CBM2 domain, as in both mutants, binding to BC is significantly reduced relative to WT (Fig. 4a). Though similar in size, the mutant with just the CD of *Tf*AA10B has much less binding affinity than WT *Tf*AA10B. This is due to the presence of the additional CBM2 domain of *Tf*AA10B, which provides additional binding to regions of highly crystalline cellulose at the pH optimum for activity. Based on the results presented in Figs. 4a and 5, domain removal has a significant effect on *Tf*AA10B activity [15]. The activity decreases over time similar to the nonlinear kinetics of hydrolytic cellulases [15]. Compared to the hydrolytic endocellulase, *Tf*Cel5A, *Tf*AA10B releases fewer soluble products (Fig. 5).

The activity of LPMO domain constructs shows that whereas the two LPMO catalytic domains vary in binding ability, the activity of the *T. fusca* type I LPMO benefits significantly from the CBM2 domain. The AA10B truncation mutant lacking the intermediate X1 domain shows activity on BC that is unchanged relative to WT *Tf*AA10B, but that activity was reduced for both mutants lacking the CBM2 domain. This is in agreement with some observations that have reported non-catalytic or binding related roles for X1 domains [46, 47]. This outcome suggests that similar to hydrolytic cellulases, the CBM2 is important for increasing the local concentration of the catalytic domain on the substrate surface in order to perform the catalytic attack. Due to the slower turnover rate reported previously (~ 1 min^−1^), this enrichment on the substrate surface appears essential for generating sufficient oxidative cleavages for meaningful product release. This result is in agreement with previous work integrating *T. fusca* LPMO domains into cellulosome scaffolds [12]. The removal of the X1 domain had little effect, and the presence of the CBM2 domain was key to providing LPMO activity.

### Role of the X1 domain

The domain mutants of *Tf*AA10B do not indicate a clear role for the X1 (formerly FN3-like) domain of *Tf*AA10B present between the CBM and the catalytic domain (Fig. 4a, b). The removal of the X1 domain, comparing WT to ΔX1, shows no effect. Similarly, the addition of the X1 domain to the CD does not enhance binding or activity (Figs. 4a, 5). Our results clearly show that the binding of *Tf*AA10B is mediated mainly by the CBM2 domain and to some extent by the CD. The effect of X1 deletion is mirrored in activity results, where removal has no effect. Similarly, the removal of the X1 domain had little effect on TfAA10B incorporated into scaffolds [12]. Although X1 domains are abundant in both LPMO and cellulase genes, the X1 domain is currently a domain of unknown function [48]. In some hydrolytic cellulases, such as *Tf*Cel48A and in chitinases, found in subfamily A and B of Family 18, they can be present in multiple copies [49]. X1 domains are especially abundant in extremophile amylopullulanase [50]. Whereas X1 domains have little effect in LPMOs under tested conditions, their removal from a processive endocellulase significantly reduced activity on multiple substrates [51]. Also, X1 domain removal from CbhA caused activity reduction to 50% [52]. The structure of the *Ct*CbhA X1 domains do not indicate a clear role and were shown to not destabilize cellulose structure [47]. Only two *T. fusca* cellulases, Cel5A and Cel6B, lack an X1 domain between their CD and CBM.

There have been multiple hypotheses describing the role of X1 domains, including linker protection and extension. The trend of location of X1 domains between the catalytic domain and CBMs in many LPMO genes suggests a role related to mediating binding, possibly replacing glycosylated fungal linkers or protecting long unstructured regions from proteolysis. The position of X1 domains between the CD and CBM2 domains suggest that it may play a role similar to the CBM to assist in direct binding to cellulose, which would replace glycosylated linkers as found in *Tr*Cel7A [53]. The most likely role of X1 domains in bacterial systems is to provide resistance to proteolysis, a structural feature protecting the otherwise unstructured linker between the CD and CBM domains.

Alternatively, unfolding of the X1 domain, similar to the role of the domain in mammalian titin, may give the CD more access to substrate further from the bound CBM. Forced unfolding of X1 domain via SMD has been explored [54]. The absence of disulfide bonds, which are present in the adjacent CBM2, results in less stability. This role is likely shared with the X1 domains found in other *T. fusca* hydrolyses, suggesting similar optimization of domain arrangements of bacterial LPMOs and cellulases. The X1 domain may play a more significant role in processive enzymes or in cases where substrate access for the CD is impaired. This supports the concept that the search and engagement to a free chain end is a limiting step for highly active exocellulases. The effect of X1 domain deletion in LPMOs may not be evident under current experimental conditions, due to the abundance of substrate.

## Conclusions

In this work, the two LPMOs of *Thermobifida fusca* were explored through deletion of the multiple domains of *Tf*AA10B and mutagenesis of surface residues of *Tf*AA10A (H37A, W82A, N83A and Y213F). The crystal structure of *Tf*AA10A was solved, showing significant similarities with other type III bacterial LPMOs. The activity of the LPMO mutants was measured on crystalline cellulose and characterized using short time course kinetics [15, 55].

The structural features of the *Tf*AA10A were in agreement with other known bacterial LPMOs [16]. Electron density at the active sites of the two molecules in the asymmetric unit was weak and copper coordination was not perfect but the positions of the copper atoms and the residues of interest around the flat substrate binding area could be assigned without doubt. The coppers of both active sites were photo-reduced with the active site of chain A showing type I copper coordination while chain B active site retained some features of type II.

The point mutation experiments support existing models of LPMO binding and activity [31], showing that polar surface residues were likely selected for enhanced binding and the aromatic residues are important for positioning the substrate near the active site copper ion. Specifically, our results show that both Trp82 and Asn83 are important for binding but only Trp82 has a clear effect on activity. The Y213F mutation that mirrors *Tf*AA10B and fungal LPMOs had a very small effect on both binding and activity. This suggests that the additional hydroxyl group from the tyrosine affects the hydrogen bonding network near the active site copper but does not completely inhibit the activation of the copper.

The *Tf*AA10B domain truncation mutants showed that CBM2 is important for the function of the enzyme but the X1 module does not affect binding or activity. This indicates that the role of CBM2 is to increase the local concentration of the catalytic domain on the substrate surface. The importance of the CBM2 module is emphasized by the very low binding shown for the CD-only construct. The role of the X1 domain has not been conclusively established, but its presence between the CD and CBM domains suggests that it gives increased substrate access on crowded surface [47] and also acts as a proteolysis-resistant linker [53].

The role of LPMOs and the variability of abundance in genomes is not fully explored. LPMOs likely perform initial attacks into crystalline cellulose to allow larger processive cellulases to bind and attack, but the precise nature of their synergistic behavior remains to be definitively characterized.

## Additional file

**Additional file 1: Figure S1.** HPLC chromatograms of the full range of glucose gluconolactone ratios A) that demonstrate sample neutral/oxidized product ratio using B) linear standard curves for neutral and C1 oxidized monosaccharides for quantification of LPMO products.

## Abbreviations

LPMO: lytic polysaccharide monooxygenase
CBM: carbohydrate binding module
CDH: cellobiose dehydrogenase

## References

[1] Lynd LR, Weimer PJ, Willem HVZ, Pretorius IS (2002). Microbial cellulose utilization: fundamentals and biotechnology. Microbiol Mol Biol Rev.

[2] Lombard V, Golaconda Ramulu H, Drula E, Coutinho PM, Henrissat B (2014). The carbohydrate-active enzymes database (CAZy) in 2013. Nucleic Acids Res..

[3] Levasseur A, Drula E, Lombard V, Coutinho PM, Henrissat B (2013). Expansion of the enzymatic repertoire of the CAZy database to integrate auxiliary redox enzymes. Biotechnol Biofuels.

[4] Cannella D, Jørgensen H (2014). Do new cellulolytic enzyme preparations affect the industrial strategies for high solids lignocellulosic ethanol production?. Biotechnol Bioeng.

[5] Hemsworth GR, Johnston EM, Davies GJ, Walton PH (2015). Lytic polysaccharide monooxygenases in biomass conversion. Trends Biotechnol.

[6] Forsberg Z, Vaaje-Kolstad G, Westereng B, Bunæs AC, Stenstrøm Y, MacKenzie A (2011). Cleavage of cellulose by a CBM33 protein. Protein Sci.

[7] Kim S, Ståhlberg J, Sandgren M, Paton RS, Beckham GT (2014). Quantum mechanical calculations suggest that lytic polysaccharide monooxygenases use a copper-oxyl, oxygen-rebound mechanism. Proc Natl Acad Sci USA..

[8] Li X, Beeson WT, Phillips CM, Marletta MA, Cate JHD, Cate JHD (2012). Structural basis for substrate targeting and catalysis by fungal polysaccharide monooxygenases. Structure.

[9] Phillips CM, Beeson WT, Cate JH, Marletta MA (2011). Cellobiose dehydrogenase and a copper-dependent polysaccharide monooxygenase potentiate cellulose degradation by *Neurospora crassa*. ACS Chem Biol.

[10] Beeson WT, Phillips CM, Cate JHD, Marletta MA (2012). Oxidative cleavage of cellulose by fungal copper-dependent polysaccharide monooxygenases. J Am Chem Soc.

[11] Moser F, Irwin D, Chen S, Wilson DB (2008). Regulation and characterization of* Thermobifida fusca* carbohydrate-binding module proteins E7 and E8. Biotechnol Bioeng.

[12] Arfi Y, Shamshoum M, Rogachev I, Peleg Y, Bayer E (2014). Integration of bacterial lytic polysaccharide monooxygenases into designer cellulosomes promotes enhanced cellulose degradation. Proc Natl Acad Sci USA.

[13] Forsberg Z, Mackenzie AK, Sørlie M, Røhr ÅK, Helland R, Arvai AS (2014). Structural and functional characterization of a conserved pair of bacterial cellulose-oxidizing lytic polysaccharide monooxygenases. Proc Natl Acad Sci USA.

[14] Walton PH, Davies GJ (2016). On the catalytic mechanisms of lytic polysaccharide monooxygenases. Curr Opin Chem Biol.

[15] Kostylev M, Wilson D (2013). Two-parameter kinetic model based on a time-dependent activity coefficient accurately describes enzymatic cellulose digestion. Biochemistry.

[16] Frandsen KEH, Lo Leggio L (2016). Lytic polysaccharide monooxygenases: a crystallographer’s view on a new class of biomass-degrading enzymes. IUCrJ..

[17] Vaaje-Kolstad G, Houston DR, Houston DR, Riemen A, Eijsink VGH, van Aalten DMF (2005). Crystal structure and binding properties of the Serratia marcescens chitin-binding protein CBP21. J Biol Chem.

[18] Frandsen KEH, Simmons TJ, Dupree P, Poulsen J-CN, Hemsworth GR, Ciano L (2016). The molecular basis of polysaccharide cleavage by lytic polysaccharide monooxygenases. Nat Chem Biol.

[19] Winn MD, Ballard CC, Cowtan KD, Dodson EJ, Emsley P, Evans PR (2011). Overview of the CCP4 suite and current developments. Acta Crystallogr Sect D Biol Crystallogr.

[20] Dauter Z, Dauter M, Rajashankar KR (2000). Novel approach to phasing proteins: derivatization by short cryo-soaking with halides. Acta Crystallogr Sect D Biol Crystallogr.

[21] Skubák P, Pannu NS (2013). Automatic protein structure solution from weak X-ray data. Nat Commun..

[22] Cowtan K (2006). The Buccaneer software for automated model building. 1. Tracing protein chains. Acta Crystallogr Sect D Biol Crystallogr..

[23] Murshudov GN, Skubák P, Lebedev AA, Pannu NS, Steiner RA, Nicholls RA (2011). REFMAC5 for the refinement of macromolecular crystal structures. Acta Crystallogr Sect D Biol Crystallogr.

[24] Emsley P, Lohkamp B, Scott WG, Cowtan K (2010). Features and development of Coot. Acta Crystallogr Sect D Biol Crystallogr.

[25] Adams PD, Afonine PV, Bunkóczi G, Chen VB, Davis IW, Echols N (2010). PHENIX: a comprehensive Python-based system for macromolecular structure solution. Acta Crystallogr D Biol Crystallogr.

[26] Chen VB, Arendall WB, Headd JJ, Keedy DA, Immormino RM, Kapral GJ (2010). MolProbity: all-atom structure validation for macromolecular crystallography. Acta Crystallogr Sect D Biol Crystallogr.

[27] Engh RA, Huber R (1991). Accurate bond and angle parameters for X ray protein structure refinement. Acta Crystallogr Sect A..

[28] Kostylev M, Alahuhta M, Chen M, Brunecky R, Himmel ME, Lunin VV (2014). Cel48A from* Thermobifida fusca*: structure and site directed mutagenesis of key residues. Biotechnol Bioeng.

[29] Zor T, Selinger Z (1996). Linearization of the Bradford protein assay increases its sensitivity: theoretical and experimental studies. Anal Biochem.

[30] Cannella D, Hsieh CWC, Felby C, Jørgensen H (2012). Production and effect of aldonic acids during enzymatic hydrolysis of lignocellulose at high dry matter content. Biotechnol Biofuels.

[31] Wu M, Beckham GT, Larsson AM, Ishida T, Kim S, Payne CM (2013). Crystal structure and computational characterization of the lytic polysaccharide monooxygenase GH61D from the basidiomycota fungus Phanerochaete chrysosporium. J Biol Chem.

[32] Prlić A, Bliven S, Rose PW, Bluhm WF, Bizon C, Godzik A (2010). Pre-calculated protein structure alignments at the RCSB PDB website. Bioinformatics.

[33] Ye Y, Godzik A (2003). Flexible structure alignment by chaining aligned fragment pairs allowing twists. Bioinformatics.

[34] Hemsworth GR, Davies GJ, Walton PH (2013). Recent insights into copper-containing lytic polysaccharide mono-oxygenases. Curr Opin Struct Biol.

[35] Kjaergaard CH, Qayyum MF, Wong SD, Xu F, Hemsworth GR, Walton DJ (2014). Spectroscopic and computational insight into the activation of O2 by the mononuclear Cu center in polysaccharide monooxygenases. Proc Natl Acad Sci.

[36] Hemsworth GR, Taylor EJ, Kim RQ, Gregory RC, Lewis SJ, Turkenburg JP (2013). The copper active site of CBM33 polysaccharide oxygenases. J Am Chem Soc.

[37] Kittl R, Kracher D, Burgstaller D, Haltrich D, Ludwig R (2012). Production of four Neurospora crassa lytic polysaccharide monooxygenases in Pichia pastoris monitored by a fluorimetric assay. Biotechnol Biofuels..

[38] Loose JSM, Forsberg Z, Fraaije MW, Eijsink VGH, Vaaje-Kolstad G (2014). A rapid quantitative activity assay shows that the Vibrio cholerae colonization factor GbpA is an active lytic polysaccharide monooxygenase. FEBS Lett.

[39] Harman LS, Carver DK, Schreiber J, Mason RP (1986). One- and two-electron oxidation of reduced glutathione by peroxidases. J Biol Chem.

[40] Kracher D, Scheiblbrandner S, Felice AKG, Breslmayr E, Preims M, Ludwicka K (2016). Extracellular electron transfer systems fuel cellulose oxidative degradation. Science..

[41] Witteveen CFB, Veenhuis M, Visser J (1992). Localization of glucose-oxidase and catalase activities in *Aspergillus niger*. Appl Environ Microbiol.

[42] Scott BR, Huang HZ, Frickman J, Halvorsen R, Johansen KS (2016). Catalase improves saccharification of lignocellulose by reducing lytic polysaccharide monooxygenase-associated enzyme inactivation. Biotechnol Lett.

[43] Vaaje-Kolstad G, Westereng B, Horn SJ, Liu Z, Zhai H, Sørlie M (2010). An oxidative enzyme boosting the enzymatic conversion of recalcitrant Polysaccharides. Science..

[44] Forsberg Z, Rohr AK, Mekasha S, Andersson KK, Eijsink VG, Vaaje-Kolstad G (2014). Comparative study of two chitin-active and two cellulose-active AA10-type lytic polysaccharide monooxygenases. Biochemistry.

[45] Harris PV, Welner D, McFarland KC, Re E, Navarro Poulsen J-C, Brown K (2010). Stimulation of lignocellulosic biomass hydrolysis by proteins of glycoside hydrolase family 61: structure and function of a large, enigmatic family. Biochemistry..

[46] Adams JJ, Currie MA, Ali S, Bayer EA, Jia Z, Smith SP (2010). Insights into higher-order organization of the cellulosome revealed by a dissect-and-build approach: crystal structure of interacting clostridium thermocellum multimodular components. J Mol Biol.

[47] Brunecky R, Alahuhta M, Bomble YJ, Xu Q, Baker JO, Ding SY (2012). Structure and function of the Clostridium thermocellum cellobiohydrolase A X1-module repeat: enhancement through stabilization of the CbhA complex. Acta Crystallogr Sect D Biol Crystallogr.

[48] Beckham GT, Bomble YJ, Bayer E, Himmel ME, Crowley MF (2011). Applications of computational science for understanding enzymatic deconstruction of cellulose. Curr Opin Biotechnol..

[49] Saito A, Fujii T, Miyashita K (2003). Distribution and evolution of chitinase genes in Streptomyces species: involvement of gene-duplication and domain-deletion. Int J Gen Mol Microbiol..

[50] Lin HY, Chuang HH, Lin FP (2008). Biochemical characterization of engineered amylopullulanase from Thermoanaerobacter ethanolicus 39E-implicating the non-necessity of its 100 C-terminal amino acid residues. Extremophiles.

[51] Zhou W, Irwin DC, Escovar-Kousen J, Wilson DB (2004). Kinetic studies of* Thermobifida fusca* Cel9A active site mutant enzymes. Biochemistry.

[52] Kataeva IA, Seidel RD, Shah A, West LT, Li XL, Ljungdahl LG (2002). The fibronectin type 3-like repeat from the *Clostridium thermocellum* cellobiohydrolase CbHa promotes hydrolysis of cellulose by modifying its surface. Appl Environ Microbiol..

[53] Payne CM, Resch MG, Chen L, Crowley MF, Himmel ME, Taylor LE (2013). Glycosylated linkers in multimodular lignocellulose-degrading enzymes dynamically bind to cellulose. Proc Natl Acad Sci USA..

[54] Craig D, Krammer A, Schulten K, Vogel V (2001). Comparison of the early stages of forced unfolding for fibronectin type III modules. Proc Natl Acad Sci USA..

[55] Shu Z, Wang Y, An L, Yao L (2014). The slowdown of the endoglucanase *Trichoderma reesei* Cel5A-catalyzed cellulose hydrolysis is related to its initial activity. Biochemistry.

