# Supplementary material for: Structure of a Thermobifida fusca lytic polysaccharide monooxygenase and mutagenesis of key residues
Source: Biotechnol Biofuels. 2017 Nov 30;10:243. doi: 10.1186/s13068-017-0925-7 (PMC5708082; doi:10.1186/s13068-017-0925-7)
Supplement: Supplementary file 1 — Additional file 1: Figure S1. HPLC chromatograms of the full range of glucose gluconolactone ratios A) that demonstrate sample neutral/oxidized product ratio using B) linear standard curves for neutral and C1 oxidized monosaccharides for quantification of LPMO products. [file 13068_2017_925_MOESM1_ESM.docx]

**Additional information for: Structure of a *Thermobifida fusca* lytic polysaccharide**

**monooxygenase and mutagenesis of key residues**

Nathan Kruer-Zerhusen^1^, Markus Alahuhta^2^, Vladimir V. Lunin^2^, Michael E. Himmel^2^,

Yannick J. Bomble^2*^, and David B. Wilson^1^

Additional file: Figure S1.


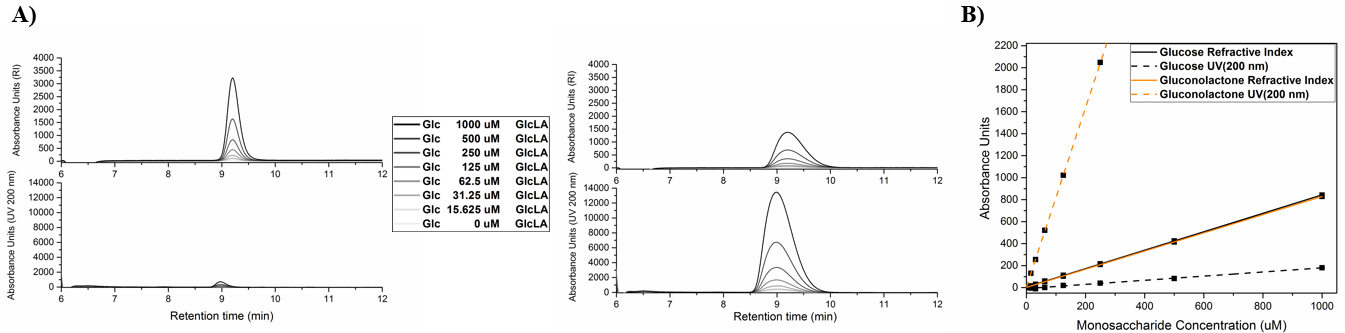


Supplementary figure S1: HPLC chromatograms of the full range of glucose gluconolactone ratios A) that demonstrate sample neutral/oxidized product ratio using B) linear standard curves for neutral and C1 oxidized monosaccharides for quantification of LPMO products.
